# Field evaluation of the gut microbiome composition of pre-school and school-aged children in Tha Song Yang, Thailand, following oral MDA for STH infections

**DOI:** 10.1371/journal.pntd.0009597

**Published:** 2021-07-26

**Authors:** Katharina Stracke, Poom Adisakwattana, Suparat Phuanukoonnon, Tippayarat Yoonuan, Akkarin Poodeepiyasawat, Paron Dekumyoy, Kittipong Chaisiri, Alexandra Roth Schulze, Stephen Wilcox, Harin Karunajeewa, Rebecca J. Traub, Aaron R. Jex

**Affiliations:** 1 The Walter and Eliza Hall Institute of Medical Research, Melbourne, Victoria, Australia; 2 Department of Medical Biology, The Faculty of Medicine, Dentistry and Health Sciences, The University of Melbourne, Melbourne, Victoria, Australia; 3 Department of Helminthology, Faculty of Tropical Medicine, Mahidol University, Bangkok, Thailand; 4 Social and Environmental Medicine, Faculty of Tropical Medicine, Mahidol University, Bangkok, Thailand; 5 Department of Medicine–Western Health, The University of Melbourne, Melbourne, Victoria, Australia; 6 Department of Veterinary Biosciences, Faculty for Veterinary and Agricultural Sciences, The University of Melbourne, Melbourne, Victoria, Australia; University of Utah, UNITED STATES

## Abstract

Soil-transmitted helminths, such as roundworms (*Ascaris lumbricoides*), whipworms (*Trichuris trichiura*) and hookworms (*Necator americanus* and *Ancylostoma* spp.), are gastrointestinal parasites that occur predominantly in low- to middle-income countries worldwide and disproportionally impact children. Depending on the STH species, health status of the host and infection intensity, direct impacts of these parasites include malnutrition, anaemia, diarrhoea and physical and cognitive stunting. The indirect consequences of these infections are less well understood. Specifically, gastrointestinal infections may exert acute or chronic impacts on the natural gut microfauna, leading to increased risk of post-infectious gastrointestinal disorders, and reduced gut and overall health through immunomodulating mechanisms. To date a small number of preliminary studies have assessed the impact of helminths on the gut microbiome, but these studies are conflicting. Here, we assessed STH burden in 273 pre-school and school-aged children in Tha Song Yang district, Tak province, Thailand receiving annual oral mebendazole treatment. *Ascaris lumbricoides* (107/273) and *Trichuris trichiura* (100/273) were the most prevalent species and often occurred as co-infections (66/273). *Ancylostoma ceylanicum* was detected in a small number of children as well (*n* = 3). All of these infections were of low intensity (<4,999 or 999 eggs per gram for *Ascaris* and *Trichuris* respectively). Using this information, we characterised the baseline gut microbiome profile and investigated acute STH-induced alterations, comparing infected with uninfected children at the time of sampling. We found no difference between these groups in bacterial alpha-diversity, but did observe differences in beta-diversity and specific differentially abundant OTUs, including increased *Akkermansia muciniphila* and *Bacteroides coprophilus*, and reduced *Bifidobacterium adolescentis*, each of which have been previously implicated in STH-associated changes in the gut microfauna.

## Author summary

Soil-transmitted helminth infections exhibit a significant negative impact on population health worldwide. In particular in endemic regions sub-groups such as children suffer disproportionally from infection due to impoverished living conditions. Yet many of these regions have no or limited availability of infection prevalence data to inform intervention strategies. Application of low sensitivity diagnostic tools such as microscopy exacerbate this issue. Moving towards molecular tools is key in order to establish a more accurate and complete epidemiological profile that can serve as a reference standard for future studies. Recently, a number of studies investigated the microbiota as an important factor determining overall patient health. The gut microbiota can be altered through medication, lifestyle factors and co-inhabitation of the same environmental niche with STHs. However, these microbiota studies often lack a characterised baseline. The current study provides baseline epidemiology data for a cohort of pre-school and school-age Thai children using a molecular diagnostic technique. We estimate the STH infection prevalence and intensity and characterise the host gut microbiota profile at a single cross-sectional timepoint comparing infected with uninfected children. This data can serve as a starting point for future studies investigating the impact of environmental factors and parasitic infections on patient health.

## Introduction

Soil-transmitted helminths (STHs), namely roundworms (*Ascaris* spp.), whipworms (*Trichuris trichiura*) and hookworms (*Ancylostoma* spp. and *Necator americanus*), are among the most common parasitic causes of neglected tropical disease worldwide [1]. These helminthiases disproportionately affect children, in whom they cause a global estimated burden of 1.2 million disability adjusted life years (DALYs) [2]. Depending on the infective species, infection intensity and host nutritional and immunological status, symptoms include malnutrition, diarrhea, abdominal pain and anaemia, which may lead to stunting, wasting and impaired physical or cognitive development [3]. In school-aged children specifically, STH infections have been shown to cause cognitive and educational deficits [4].

Since 2012, in response to the UN Millennium Development Goals, there has been a concerted global effort to reduce STH burden in children through annual or biannual treatment with benzimidazoles treating >75% of at-risk, school-aged children in endemic regions [5]. These programs have reduced the intensity of infection and disease-related morbidity [6]. However, quantifying the magnitude of this change and its sustainability is limited by the availability of methods with adequate sensitivity to diagnose infection under conditions of low worm burden and low population prevalence [7]. As endemicity rates fall, there is a need to move from diagnostic tools such as the Kato-Katz thick smear (KKTS) method toward molecular-based methods such as quantitative PCR [8,9].

In addition to the direct consequences of infection, gastrointestinal pathogens may alter the host gut microbiome [10], and impact on bacterial diversity and abundance [11–13], brain function [14–16], digestive health [17,18], immune function [19–21], and development [17,18]. Although this impact is well studied for viral, bacterial and protistal causes of diarrhoeal disease, the effects of STH infections on the gut microbiome are not well characterised and appear complex [10]. Several studies indicate that STH increases bacterial diversity and richness [11–13]. But not all STH studies support these findings [22], and their potential pathology has not been determined [16]. One study found that gut microbiome composition was predictive of an STH infection [23]. However, ascribing cause and effect from this finding is not simple, as gut microbial composition may influence susceptibility to STH infection [24]. Additional studies of microbiome composition in helminth infections are needed. One challenge faced by such studies in highly endemic populations is that there is no data to differentiate acute from chronic effects of these parasites on gut microfauna, and little to indicate if uninfected individuals at the time of study have been affected by prior helminth infection or represent an appropriate ‘healthy control’ for microbiome characterisation.

Here, we undertook a cross-sectional study of STH infection and faecal microbiome composition in a population of pre-school and school-aged children in a remote community in Tha Song Yang district, Tak province, Thailand. Although Thailand has comparatively low national STH prevalence, the country has many rural and refugee communities, in which low-intensity infections remain highly endemic despite on-going oral MDA programs [25,26].

## Methods

### Ethics statement

Oral informed consent was received from all parents or legal guardians for participants under the age of 18 years. Ethics approval, including approval of oral informed consent, was provided by Mahidol University (FTM ECF-019-05) as well as by the Human Research Ethics Committee of the Walter and Eliza Hall Institute of Medical Research (HREC Project #16/10).

### Study area, faecal sample collection and processing

A total of 273 faecal samples from 2- to 6-year-old pre-school and school-aged children were collected from the 28^th^ to 30^th^ of March 2017 among eleven day-care centres in the Mae Song subdistrict, Tha Song Yang district, Tak province, Thailand. In addition, the local field team recorded epidemiological information for each child, such as age, sex, school, date of birth, height and weight. An overview of the entire process can be found within the supporting information files (S1 Fig). Stool collection kits consisting of a faecal collection container, disposable gloves, a zip-locked bag and study information sheet were distributed to each child’s caregiver. Within 24 hours of distribution caregivers were requested to return a double-contained stool sample (30 mL max volume). For each child a de-identified participant ID, the date and time of stool collection and processing were recorded. Stool samples were returned within an average of 12 hours and 1 gram of sample was immediately examined for STH infections by direct duplicate Kato Katz thick smear performed by two independent microscopists. The remainder of each stool sample was transferred to the laboratory field site in Mae Sot district, Tak province, Thailand within 24 hours on wet ice. There, stools were pre-processed for international transport according to their classification using the Bristol Stool Chart [27]. Type 5–7 stools were centrifuged at 14,000 x g for 5 minutes, the supernatant discarded and part of the pellet stored in DESS at room temperature (1:1 sample to preservative ratio; 20% dimethyl sulfoxide, 0.25 M ethylenediaminetetraacetic acid, saturated sodium chloride) [28]. Type 1–4 stools were stored directly in DESS without the requirement for an additional centrifugation step. Preserved stools were transported to the main laboratory located at the Department of Helminthology, Faculty of Tropical Medicine, Mahidol University (Bangkok, Thailand) within 72 hours on wet ice, where they were split into two 1–1.5 gram aliquots in 2 mL universal Nunc tubes and stored at 4°C. Cold stored DESS preserved stools were shipped to the Walter and Eliza Hall Institute of Medical Research on cold packs. Upon arrival, stool samples were long-term stored at -20°C.

### DNA extraction

Stool samples were thawed at RT, homogenized using a sterile wooden spatula and DESS preservative removed by resuspending each sample twice in 50 mL sterile water, centrifuging at 2,000 x g for 3 minutes and decanting the supernatant. Up to 150 mg of the faecal pellet was used for DNA extraction using the Bioline ISOLATE Fecal DNA Extraction kit according to the manufacturer’s instructions (Bioline, Australia). Extracted DNA was assessed for quality and quantity using the Qubit 2.0 fluorometer (Thermo Fischer Scientific, Australia).

### STH infection diagnosis and microbiome characterisation

A previously published multiplexed-tandem qPCR assay [29] was used to qualitatively and quantitatively detect STH infections among all faecal samples. We used this test to screen for roundworm (*Ascaris lumbricoides*), whipworm (*Trichuris trichiura*) and hookworm (*Ancylostoma* spp. and *Necator americanus*) species following the manufacturer’s instructions (AusDiagnostics, Ltd. Py., Australia).

The faecal microbial community for DESS preserved samples was characterised by 16S rRNA gene sequencing. Each stool sample was sequenced in duplicate using the universal bacterial 16S V4 primers (515F 5’-CTGAGACTTGCACATCGCAGCGTGYCAGCMGCCGCGGTAA-3’ and 806R 5’-GTGACCTATGAACTCAGGAGTCGGACTACNVGGGTWTCTAAT-3’) on the Illumina MiSeq platform (Illumina, USA) [30,31]. Each 96-well PCR plate contained a negative (dH_2_O) and positive (ZymoBIOMICS microbial community DNA standard, Zymo Research, Integrated Sciences, Australia) control, with individual reactions conducted under the following conditions: 10 μl OneTaq master mix with standard buffer (2x) (New England Biolabs, USA), 0.4 μl of each primer (10 μM) and 2 μl DNA template made up to a total reaction volume of 20 μl with water were amplified for 3 minutes at 95°C, 45 seconds at 95°C, 60 seconds at 50°C and 90 seconds at 72°C in 20 cycles with a final extension time of 10 minutes at 72°C. Following first round amplification all PCR products were diluted 1:5 in molecular grade H_2_O and diluted products used as a template for a second PCR amplification using unique adapter barcodes (8-mer) for each sample. Each PCR reaction was conducted under the following conditions: 15 μl OneTaq master mix with standard buffer (2x) (New England Biolabs, USA), 1.5 μl of each primer (10 μM) and 10 μl of diluted PCR product made up to a total reaction volume of 30 μl using water were amplified for 3 minutes at 95°C, 45 seconds at 95°C, 60 seconds at 55°C and 90 seconds at 72°C in 25 cycles with a final extension time of 10 minutes at 72°C. Quality control was performed using a representative selection of PCR products from all rows and columns on each plate through size separation on a 1.5% agarose gel. Following amplification, 5 μl of each product was pooled into the final sequencing library and any artifacts removed using the NucleoMag NGS clean-up and size-select kit (0.8X) as per the manufacturer’s instructions (Machery-Nagel). Pooled libraries were eluted in 55 μl of sterile H_2_O and quantified on the Agilent 4200 TapeStation and amplicon sequenced on the Illumina MiSeq platform using the MiSeq Reagent Kit V3 2 x 300bp as per the manufacturer’s instructions (Illumina, USA).

### Data analysis

#### STH Infection Prevalence and Intensity

MT-PCR data were processed in Excel 16.13.1 (Microsoft, USA), Stata 12.1 (STATA Corp, USA), visualized using GraphPad Prism 7.0d (GraphPad Software Inc., USA) and stool samples deemed infection positive or negative as per MT Analysis Software output (AusDiagnostics Pty. Ltd., Sydney). Kappa statistics (interrater reliability), sensitivity, specificity and correlation with epidemiological variables were calculated to evaluate performance of the MT-PCR method compared to the gold standard KKTS using Stata 12.1 (STATA Corp, USA). Impacts of STH infections defined as stunting and wasting were calculated as height-for-weight, height-for-age and weight-for-age z-scores compared to the 2006 WHO child growth standard [32]. Anthropometric z-score calculations were performed in Stata 12.1 (STATA Corp, USA) using the “zscores06” command [33].

#### Gut microbiome characterisation of the V4 16S rRNA gene

Following comparative analysis of three-way sample preservation as described elsewhere [34], we examined STH infection prevalence and intensity based on DESS preserved stools only. For microbiome characterisation, we tested for sequence batch effects in the initial 48 and subsequent 225 sequenced DESS preserved samples. We did this by merging both datasets before the OTU clustering step in vsearch and plotted alpha- (observed richness p-value 0.9654; inverted Simpson p-value 0.9088) and beta-diversity (p-value 0.001). Because the intra-sample diversity showed no significant change among sequencing runs, we combined the data for all subsequent analyses.

The microbial community diversity and composition was analysed using an established protocol [35]. A QIIME1 mapping file containing metadata for each replicate according to their unique index barcode was generated [36], followed by merging of paired-end reads using PEAR [37] with the following parameters: minimum overlap 100 bp, maximum assembly length 600 bp and minimum assembly length 80 bp. Resulting sequences were demultiplexed, orientation adjusted (p = 0.90, Phred quality score cut-off 29, base call accuracy>99.9%) and the 16S V4 rRNA gene universal primers trimmed (https://github.com/PapenfussLab/Jocelyn_tools/blob/master/amplicon_tools/trim_fasta_amplicons.py). Using Mothur 1.39.5 the trimmed sequences were aligned to the Silva database, the alignment cut and filtered to remove overhangs at both ends [38]. Next, sequences were deduplicated, clustered into operational taxonomy units (OTUs), a reference generated, and chimeras filtered using vsearch (minimum sequence length 64 bp). Taxonomy was assigned using the Greengenes database [39]. A phylogenetic tree was generated in QIIME1 using the make_phylogeny.py script by aligning all sequences to the reference in Mothur. Finally, the metadata, taxonomy and phylogeny information were compiled into a biom file with all quality filtered sequences. The full pipeline is available online (https://github.com/PapenfussLab/RothSchulze_microbiome).

In RStudio 1.1.453 technical replicates were merged and data (a) rarefied for alpha-diversity and differential abundance analysis, or (b) normalised using the cumulative sum squaring in QIIME1 for beta-diversity analysis, using phyloseq 1.26.1 [40]. Alpha-diversity measures were estimated by plotting observed richness, inverted Simpson and Shannon estimated diversity indexes using the plot_richness function in phyloseq 1.26.1 and visualized with microbiomeSeq 0.1 (https://github.com/umerijaz/microbiomeSeq). Statistical analysis was performed with pairwise analysis of variance (ANOVA) tests on a fitted, linear and releveled regression model on observed richness data (estimate_richness function in phyloseq 1.26.1). Relative and differential taxonomic abundance analysis were performed by estimating the mean proportion, fitting a linear model and applying a pairwise ANOVA test in RStudio 1.1.453, and the ‘DESeq2_nbinom’ algorithm in QIIME1, respectively. Beta-diversity measures were estimated using the ordinate and distance function with Bray Curtis measure for PCoA and weighted UniFrac for NMDS in phyloseq 1.26.1. Statistical analysis was performed with Euclidian distance and 999 permutations using the adonis function in the vegan package 2.5–4 and pairwise.perm.manova function in RVAideMemoire package 0.9–73. A p-value threshold of <0.05 was used for statistical significance following correction for multiple testing by the Benjamini-Hochberg false discovery rate (FDR) method for all tests. All data were visualised using the phyloseq 1.26.1 and ggplot 3.1.0 packages in RStudio 1.1.453 (RStudio, Inc., USA).

## Results

### Cohort epidemiology

All 273 participants (133 male and 132 female) included in this study were pre-school and school-aged children as follows: 3 years (18 female, 27 male), 4 years (45 female, 38 male), 5 years (35 female, 35 male), 6 years (24 female, 24 male) and 27 individuals with no age information (10 female, 9 male). Eight individuals had no sex information provided. Participants were from 11 schools from Tha Song Yang district, Tak province in Thailand, namely: Mae Song, Thi Mo Ko Tha, Mae Salid Khi, Mae Salid Luang, Mae Salid Noi, Krae Khi, Mae Kho, Mae Nil, Mae Nil Khi, Huay Manhok and Huay Manhok Khi. Stool samples were collected from the 28^th^ to 30^th^ of March 2017 as part of an ongoing local field study (S1 Table).

Child growth measures were available for 246 (90.11%, aged 0–5 years) of the 273 children with average measures as follows: mean age 59.2 completed months (36–82), mean weight 15.8 kg (8.2–21.7) and height 103.5 cm (78–122) (S2 Table). Of this subset, 27 (11.0%) children were stunted (height-for-age < -2 standard deviation from median) including 15 with any STH infection, six (2.4%) were wasted (weight-for-height < -2 standard deviation from median) including two with any STH infection and 17 (6.9%) were underweight (weight-for-age < -2 less than standard deviation from median) including nine with any STH infection. Further, four (1.6%) children were deemed too tall for their age (height-for-age > 2 standard deviation from median) including two with any STH infection and 1 (0.4%) had a weight-for-height measure above two standard deviations from median. We found no association between any of these metrices and bacterial (alpha-/beta-) diversity measures.

Total infection prevalence of any helminth species by microscopy was 49.3% by KKTS (Table 1). Infection intensity as determined by microscopic egg count ranged from 1 –>1,000 *As*. *lumbricoides* fertilized/unfertilized eggs (mean egg count 595.89) and 1–190 *T*. *trichiura* eggs (mean egg count 21.23). We note that 51 (46.8%) and 15 (17.4%) *As*. *lumbricoides* KKTS positive samples had egg densities reaching a threshold of 1,000 and 500 eggs per gram at which counting ceased, respectively (S2 Fig). Hookworm prevalence was not assessed as it was not feasible to process samples within one hour after drop-off [41]. Although not part of this study, each sample was additionally assessed for other parasitic infections via field-based microscopy, with the results as follows: 2 *Enterobius vermicularis*, 41 *Giardia duodenalis*, 5 *Entamoeba histolytica*, 69 *Entamoeba coli*, 29 *Blastocystis* spp., 5 *Iodamoeba bushlii*, 53 *Endolimax nana*, 10 *Chilomastix mesnili* and 1 *Cystoisospora bellii* infections.

**Table 1:** STH infection prevalence and intensity among SAC children from Tha Song Yang district, Tak province, Thailand including all DESS preserved samples (*n* = 273). Interrater reliability measure or kappa values are classified as follows: κ 0.0–0.2 non to slight, 0.2–0.4 fair,0.4–0.6 moderate, 0.6–0.8 substantial, 0.8–1.0 almost perfect [42]. Sensitivity is classified as the rate of true positive samples and specificity as the rate of true negative samples compared to a known gold standard (Kato Katz thick smear). Epidemiological and microscopy data for 5 individuals were missing.

*Total cohort (n = 273)*	Prevalence by KKTS (*n* = 268)	Mean total egg count	Prevalence by MT-PCR (*n* = 273)	Mean gene copy number	Mean cycle threshold value	Observed Agreement (Expected agreement) (*n* = 268)	Sensitivity	Specificity	Kappa statistic (95% CI)
Any STH infections	132 (49.3%)	-	141 (51.6%)	-	-	90.3% (50.0%)	93.2%	87.5%	0.806 (0.770–0.842)
*As*. *lumbricoides*	109 (40.7%)	595.89	107 (39.2%)	273,375	23.82	91.8% (51.9%)	89.0%	93.7%	0.829 (0.794–0.864)
*T*. *trichiura*	86 (32.1%)	21.23	100 (36.6%)	710	30.89	88.4% (54.7%)	89.5%	87.9%	0.745 (0.702–0.788)
*An*. *ceylanicum*	-	-	3 (1.1%)	3,058	28.85	-	-	-	-

#### STH infection prevalence and intensity

Based on molecular testing, STH prevalence was 39.2% (*n* = 107), 36.6% (*n* = 100) and 1.1% (*n* = 3) for *As*. *lumbricoides*, *T*. *trichiura* and *An*. *ceylanicum*, respectively (Table 1). Diagnostic target gene copy number for STH positive samples ranged from 39–4,711,749 for *As*. *lumbricoides* (mean copy number 272,931.8 and Ct value 23.8), 85–5,251 *An*. *ceylanicum* (mean copy number 3,058.3 and Ct value 28.9) and 33–6,343 *T*. *trichiura* (mean copy number 709.47 and Ct value 30.9) as determined by MT-PCR concentration (S1 Table). For the 51 *As*. *lumbricoides* samples containing >1,000 eggs per gram, target copy number ranged from 0–4,711,749.0 (mean copy number 505,851.5).

Diagnostic agreement between KKTS and MT-PCR was 91.8% and 88.4% for roundworm and whipworm. Diagnostic sensitivity (microscopy gold standard) was 89.0% for roundworm and 89.5% for whipworm with a diagnostic specificity of 93.7% and 87.9%, respectively. Interrater reliability (kappa) values ranged from 0.745 to 0.829 with confidence intervals from 0.770 to 0.864 (Table 1). Sixty-six infected individuals (46.8%) had co-infections with *As*. *lumbricoides* and *T*. *trichiura* by MT-PCR. Two individuals were infected with *As*. *lumbricoides*, *T*. *trichiura* and *An*. *ceylanicum*; and one with *T*. *trichiura* and *An*. *ceylanicum*. We found no correlation between schools and STH infection status, except for one school in Mae Kho having the significantly highest number of infected children (p-value <0.001) (S6 Table). We found no correlation between sex and STH infection status (p-values 0.281, 0.204, 0.143), MT-PCR Ct value (p-values 0.387, 0.208, 0.113) and MT-PCR gene copy number (p-values 0.799, 0.351, 0.685) for *As*. *lumbricoides*, *An*. *ceylanicum* or *T*. *trichiura*, respectively. Overall, we found a relatively even distribution of species-specific infections among all age groups: 16 *As*. *lumbricoides*, 1 *An*. *ceylanicum* and 15 *T*. *trichiura* among 3-year old’s; 30 *As*. *lumbricoides*, 0 *An*. *ceylanicum* and 28 *T*. *trichiura* among 4-year old’s; 29 *As*. *lumbricoides*, 0 *An*. *ceylanicum* and 31 *T*. *trichiura* among 5-year old’s; and 27 *As*. *lumbricoides*, 2 *An*. *ceylanicum* and 21 *T*. *trichiura* among 6-year old’s.

#### Gut microbiome characterisation

**Fig 1:**
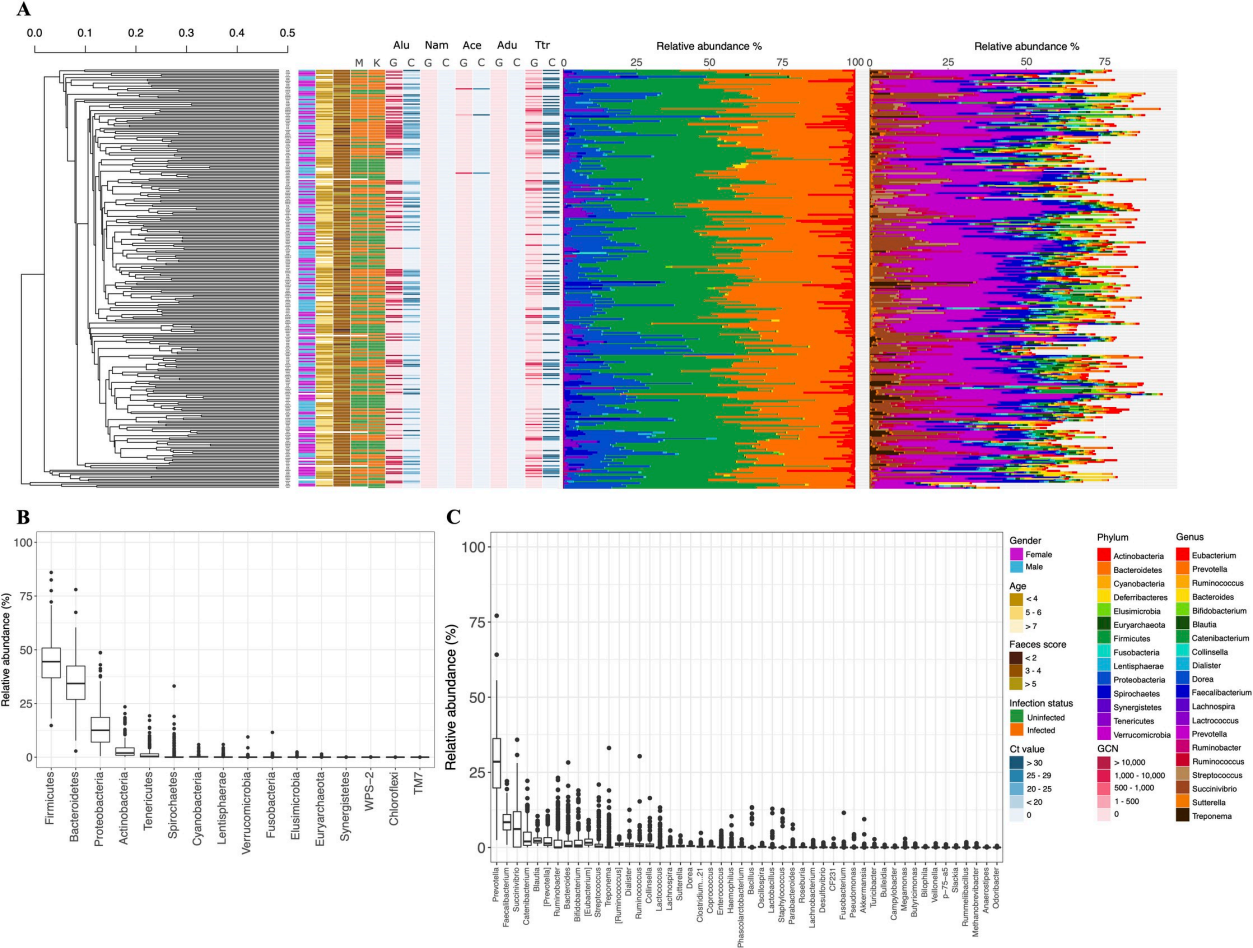
Bacterial gut community abundance to phyla and genera level, hierarchical clustering, soil-transmitted helminth infection status by microscopy (Kato Katz thick smear) and multiplexed-tandem qPCR (MT-PCR), sex, age and faecal score are depicted in the above figure (A) (adapted from Rosa *et al*. 2018 [11]). Infection intensity classification is depicted in MT-PCR gene copy number output and cycle threshold (Ct) values separated after species-specific infections: *Ascaris lumbricoides* (Alu), *Necator americanus* (Nam*)*, *Ancylostoma ceylanicum* (Ace), *Ancylostoma duodenale* (Adu) and *Trichuris trichiura* (Ttr) (from left to right). Scale bar represents evolutionary distance. Relative abundance is visualised in percentage of all bacterial phyla (B) and the top 50 bacterial genera (C). Abbreviations within the figure for schools are KK: Krae Khi, MN: Mae Nil, MNK: Mae Nil Khi, HM: Huay Manhok, HMK: Huay Manhok Khi, MSK: Mae Salid Khi, MK: Mae Kho, MSN: Mae Salid Noi, MS: Mae Song, MSL: Mae Salid Luang and TMKT: Thi Mo Ko Tha; and for diagnostics K: Kato Katz thick smear, M: MT-PCR, G: gene copy number and C: Ct value.

**Fig 2:**
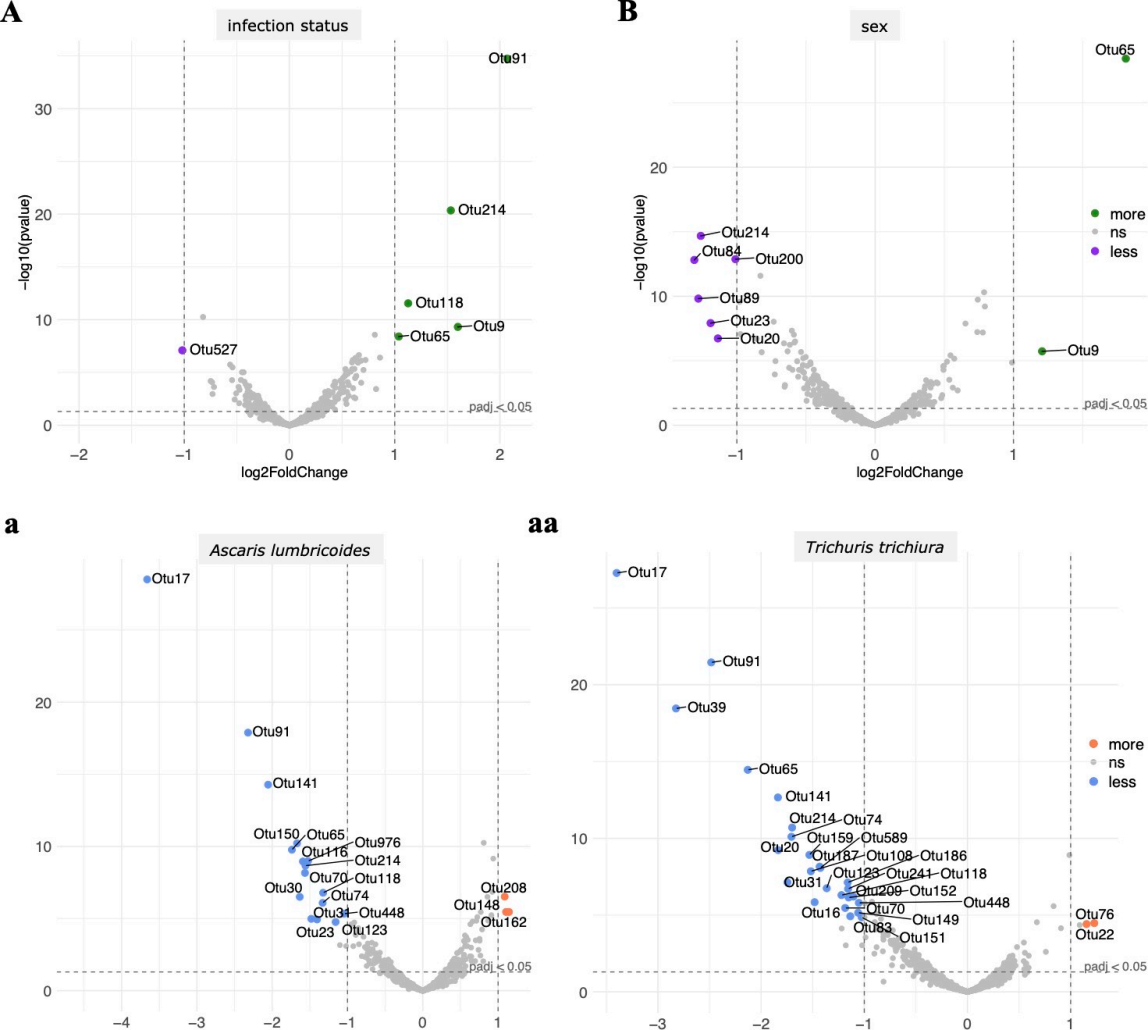
**Abundance of significantly increased and decreased operational taxonomic units (OTU) as per log fold change influenced by soil-transmitted helminth (STH) infection status estimated by multiplexed-tandem qPCR (MT-PCR) (A) for *Ascaris lumbricoides* (a) and *Trichuris trichiura* (aa) and sex (B).** Green or orange OTUs are found to be significantly more abundant and purple or blue OTUs significantly less abundant (ns = not significant). For STH infection status (A) OTU 91 *Akkermansia muciniphila*, OTU 214 *Prevotella*, OTU 65 *Bacteroides coprophilus*, OTU 118 Ruminococcaceae, OTU 9 Tenericutes are more and OTU 527 *Bifidobacterium adolescentis* is less abundant. For sex (B) OTU 65 *Bacteroides coprophilus*, OTU 9 Mollicutes, OTU 214 *Prevotella*, OTU 84 *Parabacteroides*, OTU 89 *Bacteroides uniformis*, OTU 23 *Bacteroides fragilis* are more and OTU 200 *Ruminococcus*, OTU 20 *Lactococcus garvieae* are less abundant.

**Fig 3:**
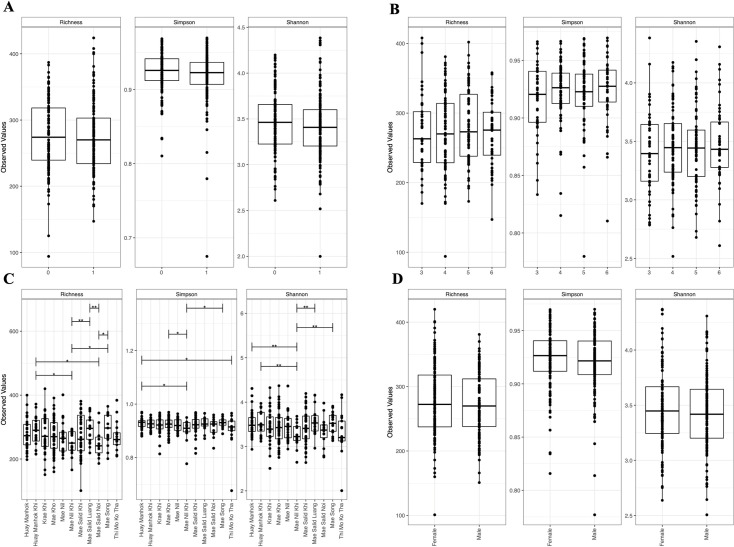
Alpha-diversity measures with analysis of variance (ANOVA) statistics of the complete cohort of faecal samples preserved in DESS (*n* = 273). Statistical analysis was performed using a p-value cut-off of 0.05 (*0.05 **0.005 ***0.0005) for soil-transmitted helminth (STH) infection status as estimated by multiplexed-tandem qPCR (MT-PCR) (0 uninfected, 1 infected) (A), age (3 to 6 years) (B), school (C) and sex (female, male) (D).

**Fig 4:**
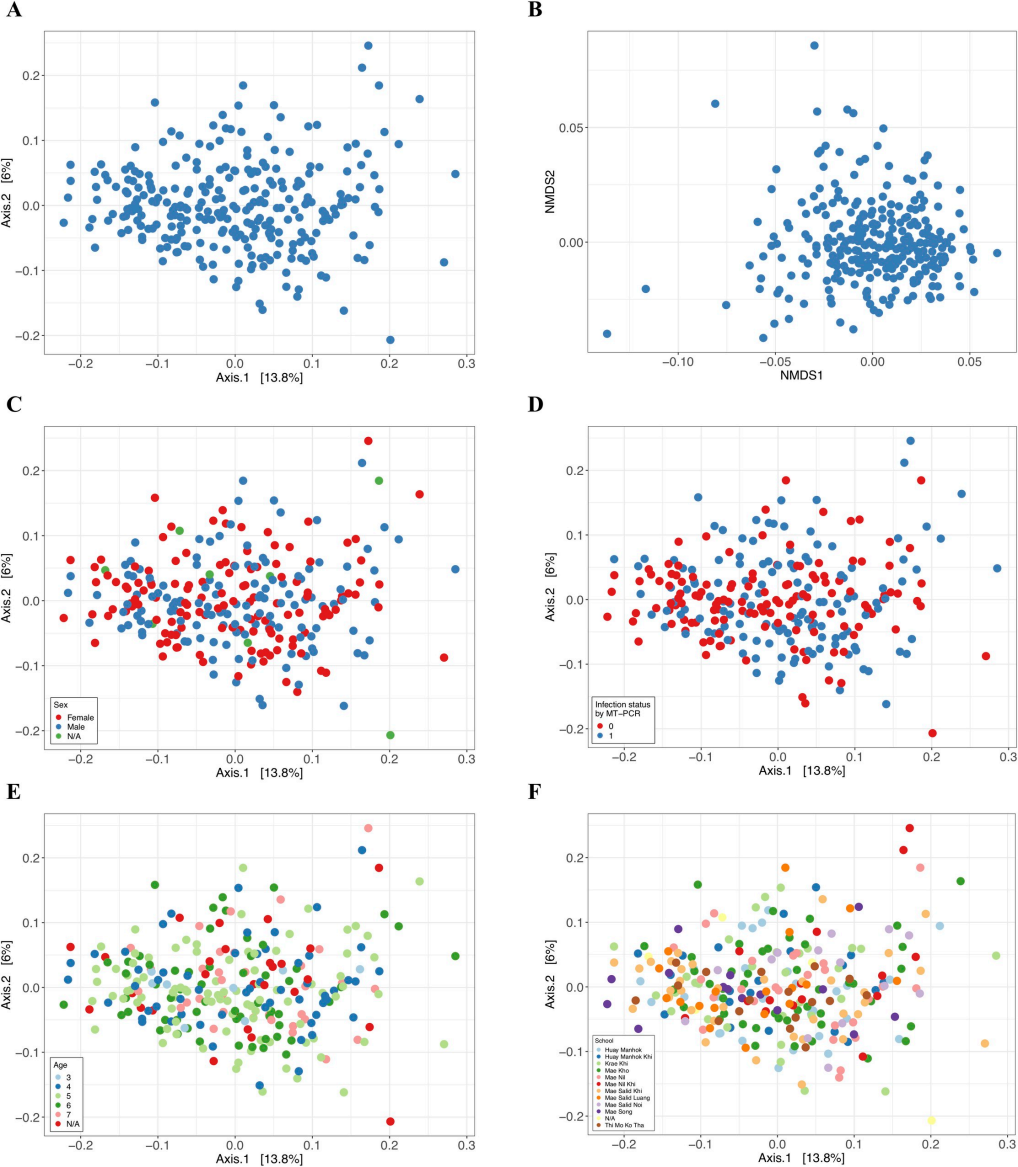
**Beta-diversity measure as calculated by principal coordinates analysis (PCoA) with Bray-Curtis distance clustering (A) and non-metric multi-dimensional scaling (NMDS) using weighted UniFrac distance measure (B) of the complete cohort of faecal samples preserved in DESS (*n* = 273).** PCoA with Bray-Curtis distance clustering for sex (C), soil-transmitted helminth (STH) infection status (D), age (E) and school (F) are shown (p-values 0.571; 0.021; 0.778; 0.032).

We explored the acute effects of STH infections on gut microbial health comparing children with (“infected”; *n* = 141) and without (“uninfected”; *n* = 132) STH infection at the time of sampling. Information on the prior infection history of children in our study was not available, and we cannot make inference on the longer-term impacts that STH infection may have on microbial development in the gut. Overall, Firmicutes, Bacteriodetes, Proteobacteria and Actinobacteria were the most abundant bacterial phyla across the complete cohort (Fig 1 and S3 Table). The majority (56.5%) of operational taxonomic units (OTUs) assigned to bacterial genera were classified as *Prevotella*, *Faecalbacterium*, *Succinivibrio* and *Catenibacterium*. Before characterising differences in the microbiome associated with STH infection, we examined the data at a cohort level, considering participant sex, age and geographic location (Fig 1). We found no significant differences at a higher taxonomic level between male and female participants. However, two OTUs (OTU 65 *Bacteroides coprophilus*, OTU 9 Mollicutes) were more and six OTUs (OTU 214 *Prevotella*, OTU 84 *Parabacteroides*, OTU 89 *Bacteroides uniformis*, OTU 23 *Bacteroides fragilis*, OTU 200 *Ruminococcus* and OTU 20 *Lactococcus garvieae*) statistically significantly less abundant comparing females to males (Fig 2 and S5 Table; p-value < 0.05). Further, we observed no significant differences in alpha-diversity or beta-diversity based on participant sex and age (Figs 3 and 4 and S4 Table).

Geographical location of some schools correlated with inverted Simpson alpha-diversity, with children from Mae Nil Khi and This Mo Ko Tha having significantly lower alpha-diversity compared to children from Huay Manhok (p-value < 0.05); and children from Mae Nil Khi showing a lower alpha-diversity than those from Mae Kho and Mae Song (Fig 3). Beta-diversity also significantly differed (p-value 0.032) among schools (Fig 4 and S4 Table). These differences were apparent at a taxonomic level, with children from Huay Manhok Khi having significantly more Actinobacteria (p-value 0.0217) and those from Mae Salid Noi, Mae Nil Khi and Mae Kho having significantly less Eubacterium (p-values 0.0311, 0.0385, 0.0433).

We found no significant difference in bacterial alpha-diversity between STH infected and uninfected study participants (Fig 3 and S4 Table). However, their faecal microbiomes did differ significantly in beta-diversity (Fig 4, p-value 0.021). Although, we found no significant changes at higher taxonomic levels, there were statistically significant (p-value < 0.05) changes in differential abundance at the OTU level (S5 Table). Five OTUs, one Verrumicrobia (OTU 91, *Akkermansia muciniphila*), two Bacteriodetes (OTU 124, *Prevotella*; OTU 65, *Bacteroides coprophilus*), one Firmicutes (OTU 118, Ruminococcaceae) and one Tenericutes (OTU 9) were significantly more abundant and one OTU (OTU 527, *Bifidobacterium adolescentis*) was less abundant comparing uninfected versus infected children (Fig 2 and S5 Table). Interestingly, when comparing differential OTU abundance between only *As*. *lumbricoides* positive (n = 41) and uninfected participants we found a large number of OTUs (n = 16) to be less abundant compared to three more abundant. The same is true for only *T*. *trichiura* positive samples (n = 33) (25 less and 2 more abundant) (Fig 2 and S5 Table).

## Discussion

In this study, we characterised the molecular STH infection status of Thai pre-school and school-aged children as well as their baseline gut microbiome profile. A large proportion of co-infections with *As*. *lumbricoides* and *T*. *trichiura* in this subpopulation is consistent with global STH-endemic estimates in other regions [43]. Using the highly sensitive MT-PCR we were able to identify three *An*. *ceylanicum* infections that were not detected by microscopy. We investigated the association between infection status and health metric (weight, height), but found no significant difference to uninfected children.

We also explored the acute effects of current STH infection on the faecal microbiome. Overall, the predominant bacterial phyla, with Bacteriodetes, Firmicutes, Proteobacteria and Actinobacteria being most abundant, were consistent with similar studies of enteric bacteria elsewhere [44–46]. A multitude of factors are able to shape the gut microbiome composition. Besides infection [10,47], this can include diet [48], age [49], caesarean versus vaginal birth [50], medication, particularly antibiotic usage [51], and numerous other factors [52]. In our study, we noted statistically significant differences in alpha-diversity with school location, possibly reflecting dietary differences. Noting these host factors, we found no evidence for an association between sex, age or school and STH infection and thus, are able to consider the effect of infection despite the confounding influence they may have had on microbial composition. Although a small proportion of children were stunted, wasted and underweight for their age, we found this did not impact microbial diversity compared to healthy children.

Numerous studies have explored the impact of STH infections on the gut microbial community, and in turn on the host immune system [53,54], child development and the risk of chronic post-infectious gastrointestinal disorders, as has been seen for parasitic protists, such as *Giardia* [55]. The results of these studies are highly variable, have been reviewed in detail elsewhere [56,57], and highlight the complexity of this topic. The lack of uninfected, community-based controls, or rather, how these controls are defined is one major challenge. In our study, we assessed the faecal microbiome of children with, at the time of sampling, patent STH infections and compared this to samples collected from uninfected children from the same community and at the same point in time. However, it is important to recognize that our observations are based on a snapshot in time and the prior infection history of our study participants is not available. This point is important in considering current studies on this field. Studies of helminth infection in mouse models have found the most consistent evidence for helminth-induced reprofiling of the gut microbial community [56], showing a loss in alpha-diversity and changes in the relative abundance of key microbial phyla, including reductions in Firmicutes and Bacteroidetes, and statistically significant shifts in abundance of a variety of specific OTUs. Studies comparing experimentally infected human volunteers to uninfected controls in non-endemic regions generally yield comparable results, showing altered alpha-diversity, species richness and the relative abundance of specific taxa, such as species of *Bacteroides* and *Turibacter* [57–59]. However, to date, field-based studies using cross-sectional surveillance, have produced more variable results [56,57], which are likely affected by the STH species and other complex biological, technical and environmental factors [57]. Recently, field studies conducted in Malaysia, Liberia and Indonesia found STH infections correlated with an increase in the host gut microbial diversity and richness [11,12], but another in Ecuador found no effect [22].

Here, we found no difference in alpha-diversity or relative abundance with STH infection. This is largely consistent with other field-based studies [56,57]. We did see differences in bacterial beta-diversity with STH infection. This too is consistent with a previous study of STH infections in Sri Lanka [46], but has not been observed in other field studies [22,60], and may depend on the infective STH species [57]. We did not see major changes in the relative abundance of bacterial phyla, but we did see specific changes in OTU composition. In our study, STH infection was associated with a statistically significant increase in abundance of *Akkermansia muciniphila*. A recent meta-analysis of published studies on the impact of helminths on gut microbiomes identified a 3-fold increase in the relative abundance of *Akkermansia* in rodent models upon helminth infection [56]. This increase has been described also in primates infected with *T*. *trichiura* [61]. The authors of the meta-analysis [56] noted that *Akkermansia* species are associated with increased mucin degradation; it is worth noting that *Trichuris* species are known for their capacity to secrete a rich cocktail of mucin-degrading proteases [62,63]. To our knowledge, ours is the first study to note this change in humans. We also found a statistically significant decrease in *Bifidobacterium*, which is consistent with prior observations in rodent models [56]. Interestingly, we saw a statistically significant increase in the relative abundance of *Bacteroides coprophilus* with STH infection. Although a previous study noted an increase in pro-inflammatory *Bacteroides vulgatus* following deworming treatment in endemic individuals [64], recent studies of gut microbiome changes associated with inflammatory conditions, including multiple sclerosis [65], and during hepatitis-c infection [66], identify decreases in both *B*. *coprophilus* and *A*. *mucinphila* in the diseased state. Overall a reduction in OTUs correlating with *A*. *lumbricoides* and *T*. *trichiura* infections is notable. It is possible, although highly speculative, that helminths manipulate both pro- and anti-inflammatory microbes during infection as part of their well-documented efforts to manipulate the vertebrate immune response, particularly through repression of Th1/Th17 and promotion of Th2 type responses [67,68]. Clearly, this requires further study.

Much of the uncertainty associated with studies to date on the impacts of STH infection on the gut microbiome likely relates to the complexity of the interaction and technical aspects of the study design or analytical approach. As noted here, study design must consider the impact of sample collection and preservation, and our hope is that our evaluation of DESS as a low-cost preservative for STH and microbiome studies will assist in overcoming this challenge. In addition, sample size, particularly in relation to studies in remote and resource-poor populations, will affect resolution and limits study design. We have been deliberate in distinguishing our capacity to assess acute impacts of current STH infections on the gut microbiome, relative to endemic controls, from efforts to assess longer term or more universal impacts of STH infections overall. The latter requires either a complex longitudinal cohort study, or, at least, community-matched controls from non-endemic populations or with a known infection history. Appropriate matched controls with no prior history of STH infection are difficult to identify, especially in endemic regions where STH prevalence typically exceeds 50% of the population. This issue likely contributes to the lack of congruence between field-based microbiome studies of STH infections (all of which are realistically limited to observations of the acute effects of STH infections) from observations in mouse models or volunteer patients in non-endemic populations. Studies of the impact of STHs on the gut microbial community must, in our view, be equally aware of the complexity that infection intensity may play on the study outcome. Our study considers primarily low-intensity infections, and we cannot infer the potential acute or chronic consequences of higher intensity infections; although it appears reasonable to speculate that both infection intensity and frequency will influence the extent to which STH infections might cause reprofiling of the gut microfauna, as has been shown for other gastrointestinal pathogens [69].

## Supporting information

S1 Fig Stool collection workflow including sample collection, processing, storage and shipping information after the Standards for Reporting of Diagnostic Accuracy Studies (STARD) guidelines [70]. STH: soil-transmitted helminths; PSC: pre-school children; SAC: school-aged children; DESS: DMSO, EDTA and NaCl; PD: 2.5% potassium dichromate, FF: fresh frozen; MT-PCR: multiplexed-tandem qPCR.
(TIFF)

S2 Fig Statistical analysis of *Ascaris lumbricoides* positive individuals with more or less than 1,000 eggs by Kato Katz thick smear in relation to multiplexed-tandem qPCR (MT-PCR) cycle threshold (Ct) value (A) and gene copy number (GCN) (B). (TIFF)

S1 Table Metadata table including epidemiological data from Thai pre-school and school-aged children, sample collection data and STH infection prevalence and intensity data via microscopy (Kato Katz thick smear) and multiplexed-tandem qPCR. All patient samples have been de-identified using a unique sample ID. *A*. *lum*: *Ascaris lumbricoides*; *N*. *ame*: *Necator americanus*; *A*. *cey*: *Ancylostoma ceylanicum*; *A*. *duo*: *Anceylostoma duodenale*; *T*. *tri*: *Trichuris trichiura*; Conc: Concentration in gene copy number; Ct: cycle threshold value.
(XLSX)

S2 Table WHO-recommended height-for-weight, height-for-age and weight-for-age z- score analysis. Patients older than 5 years of age were not included due to no available reference standard. MT-PCR: multiplexed-tandem qPCR; Ct: cycle threshold value; BMI: body mass index.
(XLSX)

S3 Table Relative abundance data of bacterial phyla and genera including statistical analysis. (XLSX)

S4 Table Operational taxonomic unit (OTU) data table for alpha- and beta-diversity measures (*n* = 273). OTU: operational taxonomic unit; NA: not available.
(XLSX)

S5 Table Differential operational taxonomic unit (OTU) abundance analysis comparing any soil-transmitted helminth (STH) infection, *Ascaris lumbricoides* infection, *Trichuris trichiura* infection (uninfected versus infected), and sex (male versus female) (*n* = 273). (XLSX)

S6 Table Participant data per soil-transmitted helminth (STH) infection and school. *A*.*lum*: *Ascaris lumbricoides*; *T*. *tri*: *Trichuris trichiura*; *A*. *cey*: *Ancylostoma ceylanicum*; *N*. *ame*: *Necator americanus*; *A*.*duo*: *Ancylostoma duodenale*; MT-PCR: multiplexed-tandem qPCR; GCN: gene copy number; Ct: cycle threshold.
(XLSX)
