# Supplementary figures and images for: Field evaluation of the gut microbiome composition of pre-school and school-aged children in Tha Song Yang, Thailand, following oral MDA for STH infections
Source: PLoS Negl Trop Dis. 2021 Jul 26;15(7):e0009597. doi: 10.1371/journal.pntd.0009597 (PMC8341710; doi:10.1371/journal.pntd.0009597)

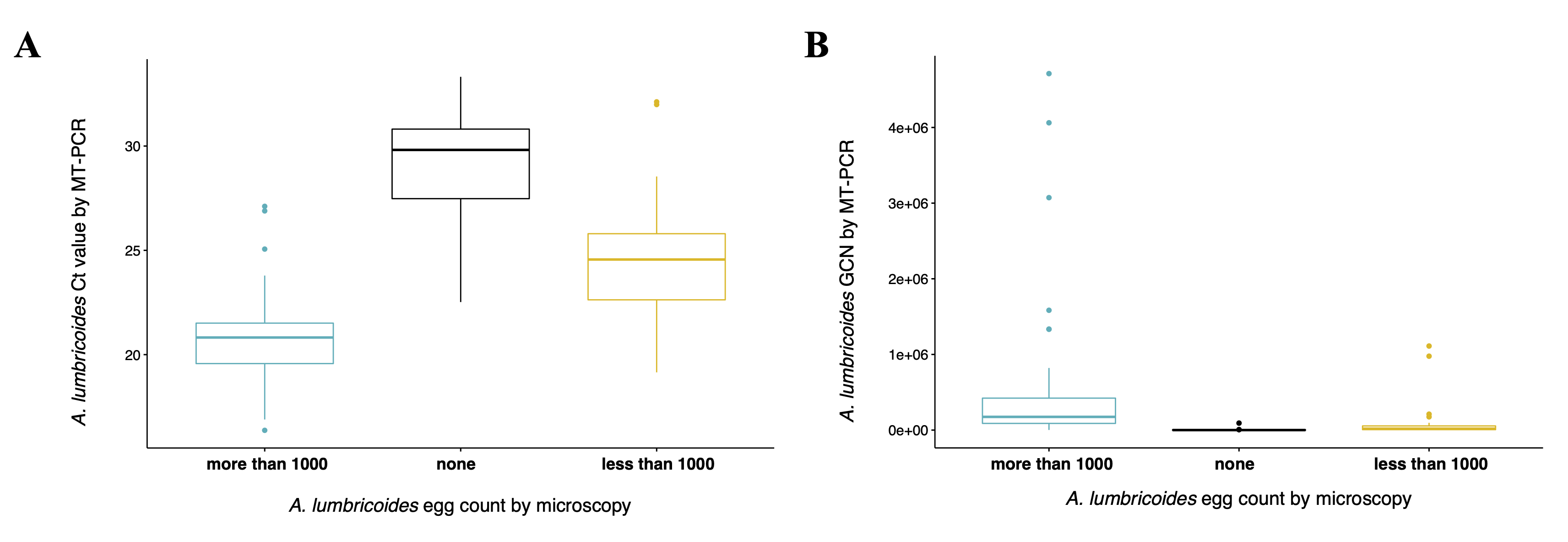

Supplement: S2 Fig — (TIFF) [file pntd.0009597.s002.tiff]
